# The cryo-thermal therapy eradicated melanoma in mice by eliciting CD4^+^ T-cell-mediated antitumor memory immune response

**DOI:** 10.1038/cddis.2017.125

**Published:** 2017-03-23

**Authors:** Kun He, Ping Liu, Lisa X Xu

**Affiliations:** 1 Med-X Research Institute, School of Biomedical Engineering, Shanghai Jiao Tong University, Shanghai, China

## Abstract

Tumor metastasis is a major concern in tumor therapy. In our previous studies, a novel tumor therapeutic modality of the cryo-thermal therapy has been presented, highlighting its effect on the suppression of distal metastasis and leading to long-term survival in 4T1 murine mammary carcinoma model. To demonstrate the therapeutic efficacy in other aggressive tumor models and further investigate the mechanism of long-term survival induced, in this study, spontaneous metastatic murine B16F10 melanoma model was used. The cryo-thermal therapy induced regression of implanted melanoma and prolonged long-term survival while inhibiting lung metastasis. It also promoted the activation of CD4^+^ CD25^−^ conventional T cells, while reduced the percentage of CD4^+^ CD25^+^ regulatory T cells (Tregs) and myeloid-derived suppressor cells (MDSCs) in the spleen, lung and blood. Furthermore, the cryo-thermal therapy enhanced the cytolytic function of CD8^+^ T cells and induced differentiation of CD8^+^ T cells into memory stem T cell (T_SCM_), and differentiation of CD4^+^ T cells into dominant CD4-CTL, Th1 and Tfh subsets in the spleen for 90 days after the treatment. It was found that good therapeutic effect was mainly dependent on CD4^+^ T cells providing a durable memory antitumor immune response. At the same time, significant increase of serum IFN-*γ* was also observed to provide an ideal microenvironment of antitumor immunity. Further study showed that the rejection of re-challenge of B16F10 but not GL261 tumor in the treated mice in 45 or 60 days after the treatment, implied a strong systemic and melanoma-specific memory antitumor immunity induced by the treatment. Thus the cryo-thermal therapy would be considered as a new therapeutic strategy to prevent tumor recurrence and metastasis with potential clinical applications in the near future.

## 

Tumor exhibits immunosuppressive state, which is responsible for its evasion of immune surveillance,^1^ resulting in tumor metastasis. Mobilizing the immune system against tumor is a promising therapeutic strategy as demonstrated in patients using immunotherapy such as anti-CTLA-4, anti-PD-1/PD-L1 antibody^2^ or CAR-T-cell therapy.^3^ Nevertheless, stimulating immune response to completely reject local tumors and distant metastasis is still far from being satisfactory, and tumor immunosuppressive microenvironment attenuates effective immune response against tumor is also illustrated.^4^ The tumor chronic inflammatory microenvironment allows the recruitment of myeloid-derived suppressor cells (MDSCs), regulatory CD4^+^ T cells (Tregs), tolerogenic dendritic cells (DCs) and tumor-associated macrophages (TAMs),^5, 6^ which are identified to generate an immunosuppressive microenvironment.^7^ Thus, induction of immune cells, such as CD4^+^ and CD8^+^ effector T cells, in a functionally hyporesponsive state are often acquired but not sufficient for mounting an efficient antitumor immune response.^8^ An effective cancer treatment is expected to destroy the tumor immunosuppression and restore normal immune surveillance to stimulate a long-lasting antitumor immune response.

Clinically, local thermal physical treatment (heating or freezing), is a common minimal invasive therapy for patients with unresectable, recurrent or metastatic tumors. It has been shown that mild or cytotoxic hyperthermia could modulate the immune system directly or indirectly.^9, 10^ Destroyed tumor tissue *in situ* following the treatment could serve as a source of tumor antigens, taken up, processed and presented by DCs to naive T cells, thus contributing to the induction of antitumor immunity.^10, 11^ Clinical reports indicate that hyperthermia induces systemic immunity to regress distant metastatic lesions spontaneously after local tumor ablation.^11, 12^ On the other hand, recent observations involved in immune response elicited by cryotherapy has been controversial, with evidence for both modulating the immune system^13^ and triggering immunosuppression.^14^ However, systemic antitumor immune response induced by hyperthermia or cryotherapy alone appears to be relatively weak, thus thermal therapeutic strategies are being explored through the combination with other therapies including immunotherapy.^15, 16, 17^

To further improve the antitumor efficacy of thermal therapy, we developed a novel therapeutic modality of the cryo-thermal therapy through application of the local rapid cooling followed by a rapid heating of tumor. As demonstrated in our previous study using the subcutaneous 4T1 murine mammary carcinoma model, the cryo-thermal therapy caused significant damage to tumor vessels and markedly enhanced tumor cell killing. Moreover, the therapy relieved immunosuppression and stimulated systemic antitumor immune response.^18, 19, 20, 21, 22^

To further study the mechanisms involved in the cryo-thermal-induced therapeutic efficacy, a murine B16 melanoma tumor model was used in this study, as its metastatic biologic characteristics are well characterized.^23^ The cryo-thermal therapy induced regression of established melanoma and prolonged long-term survival while inhibiting lung metastasis. Moreover, the cryo-thermal-induced good therapeutic effect was mainly dependent on CD4^+^ T cells orchestrating a durable, specific memory antitumor immune response. Results from this study suggested that the cryo-thermal therapy offered a new therapeutic modality to generate persistent immune memory response for tumor eradication and inhibition of tumor metastasis.

## Results

### The cryo-thermal therapy eradicated established B16F10 melanoma and prolonged long-term survival

**Figure 1:**
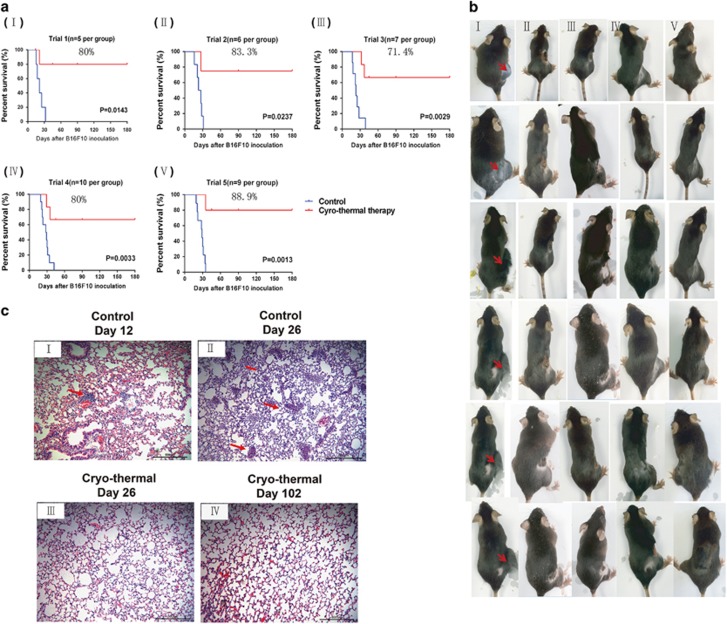
The cryo-thermal therapy eradicated B16F10 melanoma established and prolonged survival by inhibiting lung metastasis. (**a**) Kaplan–Meier survival curves in five independent trials. Kaplan–Meier survival curves were compared using log-rank tests. Survival rate in each trial was indicated; *n*=37 for control and the cryo-thermal group, respectively. (**b**)Tumor regression in the treated mice in week (I) 0, (II) 1, (III) 2, (IV) 3 and (V) 4 after the treatment. (**c**) Histological section of the lungs of the untreated mice on day 12 (I) and 26 (II), and of mice treated with the cryo-thermal therapy on day 26 (III)and 102 (IV) following tumor inoculation (red arrows indicated the metastasis lesions); × 20, scale bar, 500 *μ*m. *n*=3 per group

The cryo-thermal therapy was used to treat the primary B16F10 melanoma when the tumor volume reached about 0.2 cm^3^ on day 12 after tumor inoculation. The long-term survival rates ranged from 71.4% to 88.9% as shown in Figures 1aI–V, whereas in five control trials (*n*=37), most of the tumor-bearing mice died in 30 days after tumor inoculation. All treated primary tumors were shrunk and necrotic scabs appeared in about 2 days, usually rubbed off in 1 week and were in good health condition (Figure 1b). Therefore, the cryo-thermal therapy was effective in local tumor ablation and significantly improved survival.

Tumor cells with strong acidophilic staining, and large nuclei accumulated in the lung of mice on day 12 after tumor inoculation (Figure 1c, upper left), and more micrometastasis were distributed in the lung of untreated mice on day 26 (Figure 1c, upper right), but there were no micrometastasis found in the lung of the treated mice (Figure 1c, lower left). And 3 months after the treatment, lung tissue from the treated mice exhibited normal architectures (Figure 1c, lower right). The area of metastatic nodules in lungs was quantified by using ImagePro Plus software (Supplementary Figure 1). The results indicated that the treatment markedly inhibited metastasis, which was in a good correlation with the long-term survival.

### The cryo-thermal therapy shifted an immunosuppressive to immunostimulatory state

**Figure 2:**
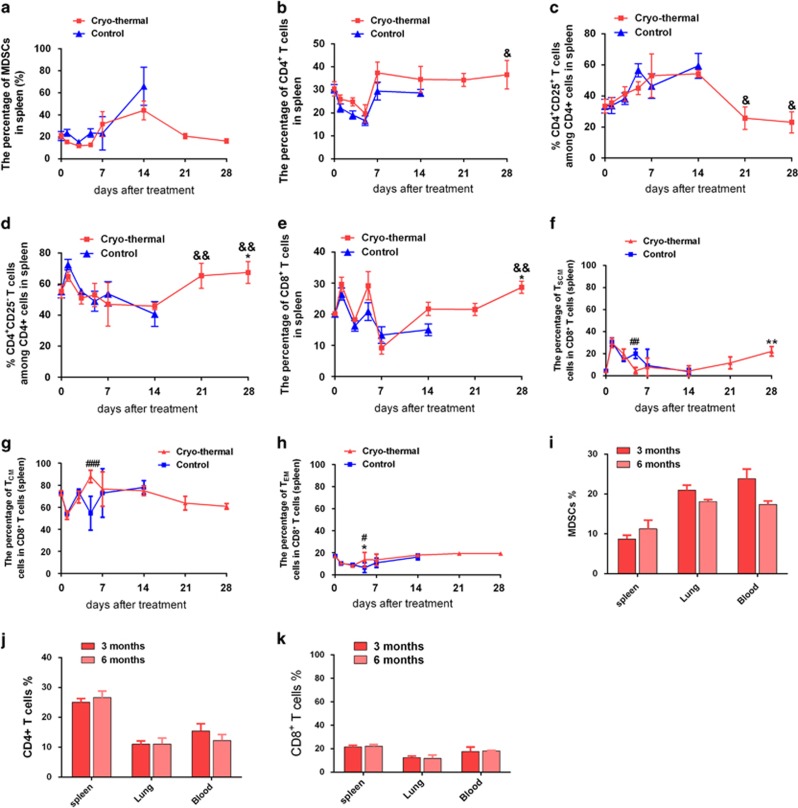
The cryo-thermal therapy shifted an immunosuppressive to immunostimulatory state. The phenotype of immune cells collected from the spleen of the treated mice on day 0, 1, 3, 5, 7, 14, 21, 28 were analyzed by flow cytometry. (**a**) The percentages of MDSCs. (**b**) The percentages of CD4^+^ T cells. (**c**) The percentages of CD4^+^CD25^+^. (**d**) The percentages of CD4^+^CD25^−^. (**e**) The percentages of CD8^+^. The percentages of T_SCM_ (**f**), T_CM_ (**g**) and T_EM_ (**h**) subsets in total CD8^+^ populations of spleen after the treatment. The percentages of MDSCs (**i**), CD4^+^ (**j**) and CD8^+^ (**k**) T cells within spleen 3 and 6 months after the cryo-thermal therapy. Data were shown as mean±S.D., *n*=3 per group. Data for bar graphs were calculated using two-way ANOVA. *P<0.05; **P<0.01 compared with baseline values. ^&^*P*<0.05; ^&&^*P*<0.01 compared with control group on day 14 after the treatment. ^#^*P*<0.05; ^##^*P*<0.01; ^###^*P*<0.001 compared with the control group on day 5 after the treatment

MDSCs are negative regulators of protective antitumor immune response and usually inhibit the activity of T-lymphocyte in cancer,^24^ having critical roles in melanoma progression.^25^ The change of MDSCs after the cryo-thermal therapy was observed. As shown in Figure 2a and Supplementary Figures 2a and b, increased level of MDSCs (up to 60%–80% in spleen, lung tissues and peripheral blood) was observed in tumor-bearing mice, but they were markedly decreased on day 14, 21, 28 after the treatment. The level of MDSCs was kept stable (10%–20%) following the treatment (Figure 2i), and close to the normal level in 3 to 6 months of observation.

CD4^+^ T cells have important roles against cancer in inducing and maintaining tumor-destructive immune responses.^26^ In this study, CD4^+^ T cells in spleen were increased from day 14–28 in the treated mice (Figure 2b), and similar results were also observed in the lung and peripheral blood (Supplementary Figures 2c and d). Moreover, the level of CD4^+^ T cells in the treated mice remained higher in the following 3 to 6 months (Figure 2j). CD4^+^CD25^−^ conventional T cells are the progenitors of populations including T helper 1(Th1), T helper 2 (Th2), T helper 17 (Th17) and follicular helper CD4^+^ T (Tfh) cells, which are essential for hosts against tumor.^27^ CD4^+^ CD25^+^ regulatory T cells (Tregs) are critical for sustaining immunological homeostasis and can inhibit antitumor responses, and contribute to immunological tolerance in cancer.^28^ In this study, the proportion of CD4^+^ CD25^+^ T cells decreased in spleen (Figure 2c), lung and peripheral blood (Supplementary Figures 2e and g), but CD4^+^ CD25^−^ conventional T cells increased from day 14 after the treatment. Although the percentages of total CD4^+^ T cells in spleen after the treatment did not seem to change from baseline values, CD4^+^ CD25^−^ conventional T-cell subset were significantly increased from baseline values on day 28 after the treatment (Figure 2d,Supplementary Figures 2f and h). The data indicated that the cryo-thermal therapy promoted the differentiation of CD4^+^ T cells to CD4^+^ CD25^−^ conventional T-cell subset on day 28 after the treatment.

CD8^+^ T cells exert cytotoxic T-lymphocyte effector function against tumor.^29^ The percentages of CD8^+^ T cells in the spleen, lung and peripheral blood of the treated mice were analyzed (Figure 2e,Supplementary Figures 2i and j). The percentages of CD8^+^ T cells in the spleen were obviously increased from baseline values on day 28 after the treatment. Taken together, these data demonstrated that the treatment relieved immunosuppression and induced a potent antitumor immune response.

**Figure 3:**
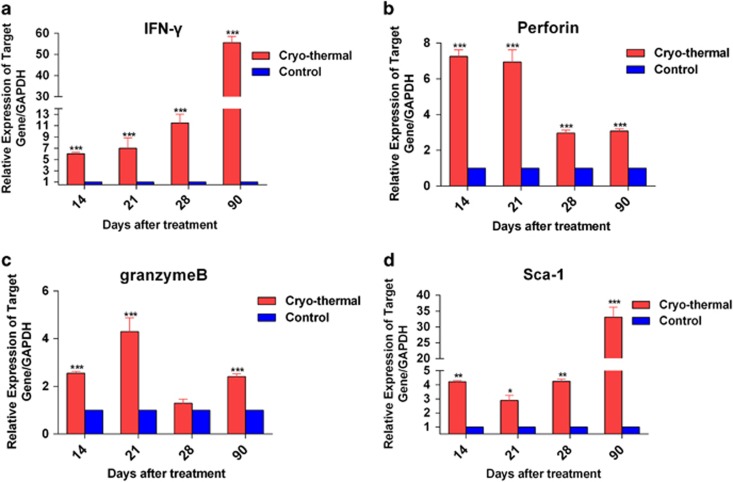
The cryo-thermal therapy enhanced memory CD8^+^ T-cell response. mRNA expression of IFN-*γ*, perforin, granzyme B and Sca-1 in splenic CD8^+^ T cells from the treated mice on day 14, 21, 28 and 90 after the treatment. The expression of IFN-*γ*, perforin, granzyme B and Sca-1 were examined by real-time PCR. The expression of IFN-*γ* (**a**), perforin (**b**), granzyme B (**c**) and Sca-1(**d**) in splenic CD8^+^ T cells from the treated mice. Data were shown as mean±S.D. Data for bar graphs were calculated using unpaired Student's *t*-test. *P<0.05; **P<0.01; ***P<0.001 in comparisons with the control group at different time points

**Figure 4:**
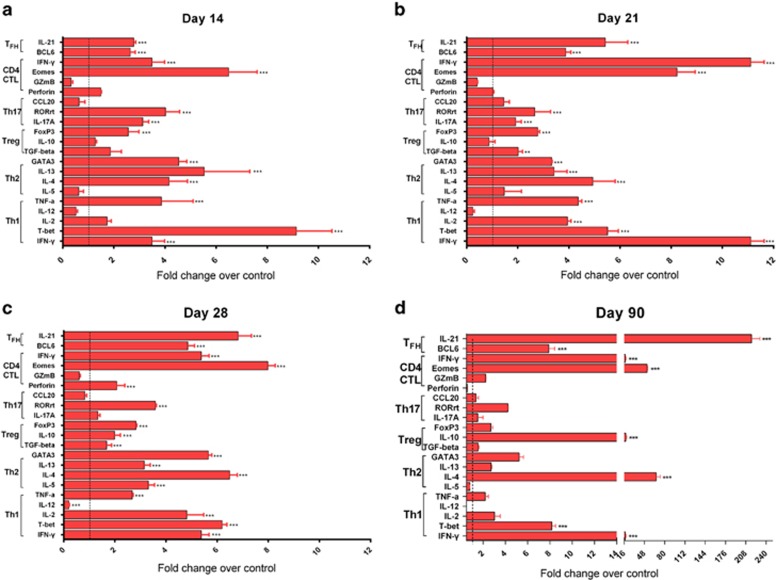
mRNA expression of marker profiles in splenic CD4^+^ T cells from the treated mice. The expression of IFN-*γ*, T-bet, IL-2, TNF-*α* (for Th1 cells); IL-5, IL-4, IL-13, GATA3 (for Th2 cells); TGF-*β*, IL-10, FoxP3 (for Treg cells); IL-17A, ROR*γ*t, CCL20 (for Th17 cells); perforin, GzmB, IFN-*γ*, Eomes (for CD4-CTL cells); Bcl-6 and IL-21 (for Tfh cells) in splenic CD4^+^ T cells from the treated mice were examined by real-time PCR. The expression of marker profiles in splenic CD4^+^ T cells on day 14 (**a**), 21 (**b**), 28 (**c**) and 90 (**d**) after the treatment. Data were shown as mean±S.D. Data for bar graphs were calculated using two-way ANOVA. **P<0.01; ***P<0.001 in comparison with the control group at different time points

Interferon gamma (IFN-*γ*) is a cytokine that elicits potent antitumor immune response by inducing Th1 response and cytotoxic T-lymphocyte (CTL) activation.^30^ The percentages of CD8^+^ T cells in the spleen were significantly increased on day 28 after the treatment (Figure 2e, *P*<0.01 compared with the control group on day 26 after tumor inoculation), along with a higher IFN-*γ* level (Figure 3a). Although the percentages of CD8^+^ T cells on day 14 and 21 after the treatment and on day 26 after tumor inoculation from tumor-bearing mice were not significantly different, the mRNA level of IFN-*γ* in splenic CD8^+^ T cells on day 14 and 21 after the treatment was obviously higher than that on day 26 after tumor inoculation, suggesting that the function of CD8^+^ T cells was changed on day 14 and 21 after the treatment, though there was no increase in the CD8^+^ T-cell populations. Similarly, the percentages of CD4^+^ CD25^−^ conventional T-cell subset in the spleen were significantly increased on day 21 and 28 after the treatment (Figure 2d, *P*<0.01 compared with the control group on day 26 after tumor inoculation), along with a higher IFN-*γ* level (Figures 4a–c). Although the percentages of CD4^+^CD25^−^ conventional T-cell subset on day 14 after the treatment and on day 26 after tumor inoculation were not significantly different, the mRNA level of IFN-*γ* in splenic CD4^+^ T cells on day 14 after the treatment was obviously higher than that on day 26 after tumor inoculation, suggesting that the function of CD4^+^ T cells was changed on day 14 after the treatment, though no increase found in CD4^+^ T-cell populations. ELISA test also identified that serum levels of IFN-*γ* was significantly elevated on day 14 after the treatment as compared with that in the control group, and IFN-*γ* level increased steadily on day 90 after the treatment (Supplementary Figure 2k). The above results showed that the cryo-thermal therapy markedly enhanced antitumor immune function of tumor-reactive CD8^+^ and CD4^+^CD25^−^ effector cells, and extremely reduced MDSCs and Tregs.

It seems that the cryo-thermal therapy stimulated the shift of an immunosuppressive to immunostimulatory state, which was more permissive for tumor eradication mediated by systemic antitumor immunity.

### The cryo-thermal therapy enhanced memory CD8^+^ T-cell response

Activated CD8^+^ effector T cells mount antitumor immune response via cytotoxic granzyme B/ perforin and by release of cytokines including IFN-*γ*. Furthermore, stem cell antigen-1 (sca-1) expression is identified as an important descriptor for long-term memory T cells.^31^ We supposed that long-term survival was attributed to memory immune response induced by the cryo-thermal therapy. The functional properties of splenic CD8^+^ T cells in the treated mice were determined by using real-time PCR. The results showed that CD8^+^ T cells significantly expressed higher levels of IFN-*γ* than that in the control from day 14 to 90 after the therapy (Figure 3a). Perforin and granzyme B transcripts in CD8^+^ T cells were also expressed at a significantly higher level following the treatment (Figures 3b and c), in comparison with the control. Further, CD8^+^ T cells from the treated mice expressed higher level of stem cells antigen-1 (Sca-1) on day 14, 21 and 28, and the highest level was observed on day 90 after the treatment (Figure 3d). These results indicated that the cryo-thermal therapy enhanced cytotoxic function of CD8^+^ T cells, and potentially promoted the formation of memory CD8^+^ T cells.

Memory T cells are further divided into effector memory T cell (T_EM_) and central memory T cell (T_CM_), and terminally differentiated to effector cells capable of sustaining a long-term protection against metastasis.^29, 31^ A new memory stem T cell (T_SCM_) is the precursor of other memory cells, and is terminally differentiated into effector cells according to the relationship T_SCM_→T_CM_→T_EM_→ effector T (E).^31^ The percentage of CD8^+^ T_SCM_ in the treated mice on day 1 and 3 exhibited the similar profile as compared with the control group (Figure 2f). On day 5, CD8^+^ T_SCM_ in spleen was significantly reduced, indicating that T_SCM_ was differentiated into central and effector memory T cells. Importantly, CD8^+^ T_SCM_ tended to increase from day 14, and significantly increased on day 28 after the treatment from baseline values (Figure 2f). The percentage of T_CM_ in the treated group on day 1 and 3 exhibited the similar profile comparable to that in the control (Figure 2g). Interestingly, on day 5, the significant increase of T_CM_ and T_EM_ in spleen was observed in the treated group (Figures 2g and h). These data demonstrated that the cryo-thermal therapy could promote and sustain a long-term protection against tumor metastasis by enhancing cytolytic function of CD8^+^ T cells and inducing differentiation of CD8^+^ T cells into T_SCM_.

### The cryo-thermal therapy promoted poly-functional polarization of CD4^+^ T-cell response

CD4^+^ T cells are differentiated into multiple sub-lineages with unique cytokine profiles that can induce and maintain destructive immune response.^26^ As shown in Figure 4a, the transcription factor T-bet of Th1 CD4^+^ T cells was elevated at least ninefold on day 14 after the treatment, with evidence of upregulated IL-2, IL-12 and TNF-*α*. The transcription factor Eomes of CD4^+^ CTL was also elevated at least sevenfold, along with upregulated IFN-*γ*. Overexpressed transcription factor ROR*γ*t of Th17 cells (fourfold) and transcription factors GATA3 of Th2 cells (4.5-fold) were also demonstrated, with evidence of upregulated Th17-associated genes (IL-17A) and Th2-associated genes (IL-4 and IL-13), respectively. Although the level of Tfh-associated genes (IL-21) and transcription factor (Bcl-6, 2.5-fold), Tregs-associated genes (IL-10 and TGF-beta) and transcription factor (FoxP3, 2.5-fold) were also significantly induced, they were at a relatively lower level as compared with Th1, CTL, Th2 and Th17 CD4^+^ T cells on day 14 after the treatment.

On day 21 after the treatment, the transcription factor Eomes of CD4^+^ CTL was most significantly increased to eightfold at the least, with evidence of upregulated IFN-*γ* (11-fold). The transcription factor T-bet of Th1 CD4^+^ T cells was elevated at least fivefold compared with that on day 14 after the treatment, along with a higher level of effector cytokines. However, the transcription factor Bcl-6 of Tfh CD4^+^ T cells was significantly upregulated (3.8-fold), as well as a higher level of effector cytokines (IL-21, 5.4-fold). Th2-associated genes (IL-4, IL-13 and IL-5) and transcription factor (GATA3, 3.3-fold) were also significantly induced. Interestingly, the Th17 transcription factor ROR*γ*t and the Tregs transcription factor FoxP3 were not significantly elevated, though their expression was higher than that in control group (2.6-fold and 2.7-fold, respectively, Figure 4b). The results indicated that the cryo-thermal therapy could promote differentiation of CD4^+^ T cells into predominant CD4^+^ CTL, Th1, Th2 and Tfh subsets from day 14 to 21 after the therapy.

On day 28 after the treatment, similar expression pattern of CD4^+^ CTL, Th1, Th2 and Tfh subsets were predominated in CD4^+^ T cells (Figure 4c). Th17 and Tregs were also significantly induced, but maintained at a certain limited level. These results suggested that effector CD4^+^ T-cell subsets were activated persistently from day 21 to 28 after the treatment.

On day 90 after the treatment, Tfh-associated gene IL-21 in CD4^+^ T cells were increased at least 200-fold, and the transcription factor Bcl-6 was also upregulated at least 7.9-fold. Interestingly, higher expression of CD4^+^CTL transcription factor Eomes was demonstrated (52-fold), as well as upregulated expression of Th1 transcription factor T-bet (eightfold). On the contrary, the expression of the transcription factor GATA3, FoxP3 and ROR*γ*t were detected at a limited level (Figure 4d). These results suggested that Tfh, CD4^+^ CTL and Th1 predominated in CD4^+^ T cells for a longer time after the treatment and potentially induced memory immunity.

Taken together, the results suggested that poly-functional polarized CD4^+^ T cells were stimulated by the treatment and dominated at different stages, and predominant CD4^+^ T- cell subsets at different phases had particular roles in promoting and sustaining the overall long-term antitumor immune response against tumor metastasis.

### Long-term survival in the treated mice was mainly dependent on CD4^+^ T cells

**Figure 5:**
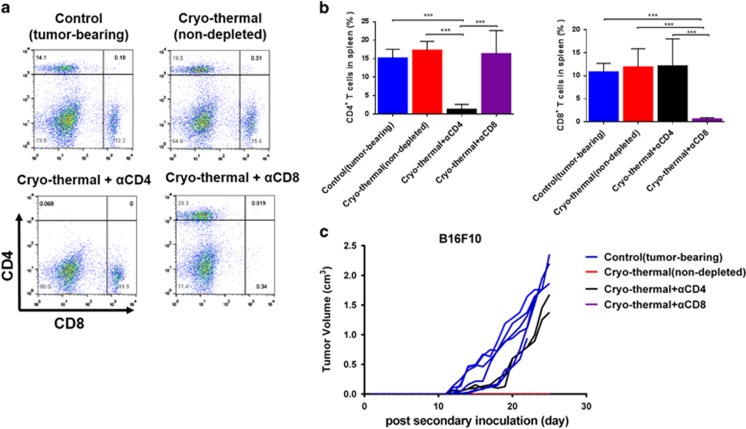
Long-term survival was mainly CD4^+^ T-cell-dependent. The treated mice on day 45 after the treatment were depleted with anti-CD4 or anti-CD8 monoclonal antibodies, respectively, then the treated mice after CD4^+^ or CD8^+^ lymphocyte depletion were re-challenged with 1 × 10^5^ B16F10 cells. (**a**) The depletion potential of each Ab was confirmed and percentages of CD3^+^ CD4^+^ and CD3^+^ CD8^+^ T cells were analyzed by flow cytometry. (**b**) Mean percentages of CD3^+^ CD4^+^ T cells and CD3^+^ CD8^+^ T cells in each group were shown (data for bar graphs were calculated using one-way ANOVA, ***P<0.001). (**c**) Tumor growth curves of individual mice in each group. Data were shown as mean±S.D., *n*=6 per group

To determine which T-cell subset was relevant to long-term survival elicited by the cryo-thermal therapy, the depletion of CD4^+^ or CD8^+^ T cells was confirmed *in vivo* (Figures 5a and b). Depletion of CD4^+^ T cells partially abolished the antitumor effect leading to tumor growth in two mice after re-challenge, which indicated that the presence of CD4^+^ T cells was crucial for mediating the antitumor memory immunity after the treatment (Figure 5c). Interestingly, all the treated mice depleted of CD8^+^ T cells completely rejected tumor growth after re-challenge. These data suggested that CD4^+^ T cells were essential to mediate antitumor memory immune response leading to long-term survival.

### The cryo-thermal therapy led to a protective tumor-specific memory response

**Figure 6:**
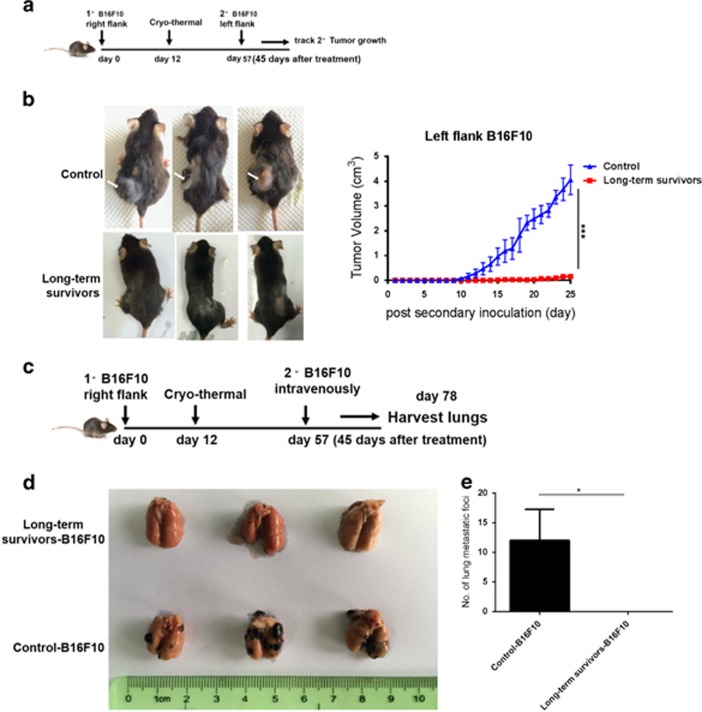
The cryo-thermal therapy protected mice from tumor re-challenge. (**a**) Schematic of experimental design. The long-term survivors were re-challenged with a subcutaneous flank injection of B16F10 cells on day 45 after the treatment. (**b**) The long-term survivors did not develop tumor after the re-challenge (left). Growth kinetics of secondary tumors were shown (right). Data were shown as mean±S.D. Tumor growth curves were analyzed using two-way ANOVA. ***P<0.001, *n*=3 per group. B16F10 melanoma cells were intravenously infused to generate lung metastasis on day 45 after the treatment, and lung metastasis were enumerated. (**c**) Schematic of experimental design. (**d**) Photographic images of lungs from long-term survivors and control mice after tumor re-challenge. (**e**) B16F10 lung metastatic tumor foci were quantified. Data were shown as mean±S.D. Data for bar graphs were calculated using Student's *t*-test. *P<0.05, *n*=3 per group

To evaluate whether long-term survivors had developed a protective antitumor memory response, survivors were re-challenged with a subcutaneous flank injection of B16F10 cells on day 45 after the treatment (Figure 6a). The age-matched naive mice were inoculated with B16F10 cells and were taken as the control group. Figure 6b displayed progressive tumor growth in all the control mice. However, all the long-term survivors did not develop tumor after the re-challenge (Figure 6b). The data further indicated that the cryo-thermal therapy mounted a systemic antitumor memory immunity to reject re-challenge.

To investigate the distal effect of the cryo-thermal therapy, primary B16F10 melanoma in C57BL/6 mice were treated with the treatment; 45 days later, B16F10 melanoma cells were intravenously infused and lung metastases were enumerated (Figure 6c). The age-matched naive mice were inoculated with B16F10 cells as the control group. All the control mice developed tumor nodules in the lungs. In contrast, the survivors were showed to entirely control distant lung metastasis (Figures 6d and e). Therefore, the cryo-thermal therapy mounted a systemic antitumor immune memory response that significantly inhibited the distal tumor growth.

**Figure 7:**
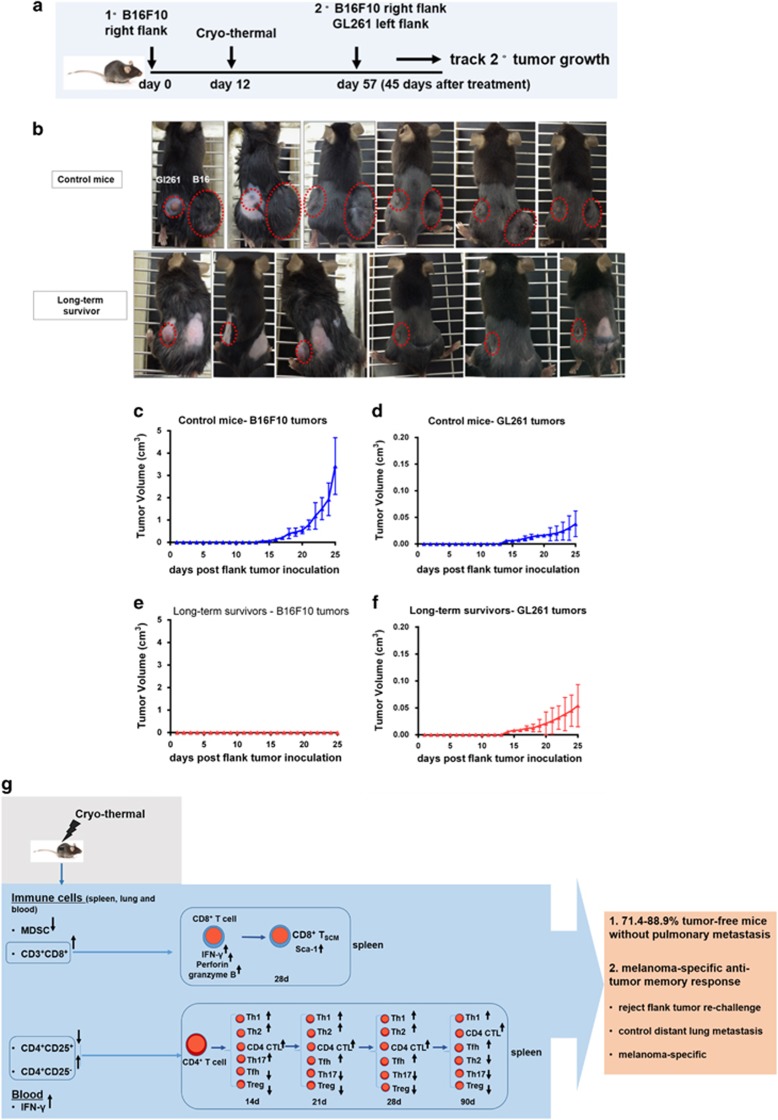
The cryo-thermal therapy resulted in a melanoma-specific memory response. (**a**) Experimental designed to determine whether the systemic antitumor memory response was specific for B16F10 melanoma tumor on day 45 after the treatment. A total 1 × 10^6^ GL261 cells were implanted subcutaneously into the left flank and 1 × 10^5^ B16F10 cells were implanted subcutaneously into the right flank. The age-matched naive mice (*n*=6) were inoculated with B16F10 cells as control (tumor-bearing) group. Tumor volumes were calculated in three dimensions. (**b**) Photographic images of tumor growth in control mice and long-term survivors (red circle indicated the palpable nodules). Control mice had progressive B16F10 (**c**) and GL261 (**d**) flank tumor growth. Long-term survivors developed a protective memory response and rejected B16F10 melanoma tumor growth (**e**), and did not affect the tumor growth of GL261 (**f**). Data were shown as mean±S.D., *n*=6 per group. (**g**) Schematic representation of the cryo-thermal therapy induced long-term tumor-free survival associated with antitumor immune memory response; 14 d, 21 d, 28 d and 90 d indicated different time points following the treatment

Next, we developed a model to determine whether the systemic antitumor memory response was specific for B16F10 melanoma. Experiments were designed to determine whether the systemic antitumor memory response was specific for B16F10 melanoma tumor on day 45 after the treatment (Figure 7a). All six control mice developed both tumor types resulting in palpable nodules on both flanks (Figures 7b–d). In contrast, only GL261 tumor grew in the treated mice with no evidence of palpable B16F10 tumor growth (Figures 7b, e and f). Furthermore, the re-challenge experiment was also performed on day 60 to add one more time point after the treatment, to rule out the elicited local antitumor immunity (Supplementary Figure 3). These results indicated that long-term survivors after the treatment developed a melanoma-specific antitumor memory response. Collectively, the cryo-thermal therapy generated strong systemic tumor-specific long-term immunologic memory capable of rejecting distant metastasis.

## Discussion

In this study, we have found that the cryo-thermal therapy not only eradicated primary melanoma without recurrence, but also inhibited lung metastasis resulting in a long-term tumor-free survival in a B16F10 melanoma mouse model. It was clearly demonstrated that the cryo-thermal therapy had an excellent efficacy profile in various aggressive tumor animal models. The treatment markedly enhanced the function of tumor-reactive effector T cells and significantly reduced immunosuppressive cells, leading to trigger-durable systemic antitumor immunity. The findings highlighted that the treatment promoted differentiation of CD4^+^ T cells into multiple CD4^+^ T-cell subsets and generated memory stem CD8^+^ T cells. Furthermore, the cryo-thermal therapy-induced durable melanoma-specific memory immune response was mainly CD4^+^ T-cell-dependent.

It is critical to induce antigen-specific cytolytic activity of CD8^+^ T cells capable of killing cancer cells.^29^ Increased CD8^+^ T cells with a high level of IFN-*γ*, perforin, granzyme B in organs and peripheral circulation of the treated mice contributed to long-term antitumor immune response. After the treatment, the reduction of CD8^+^ T_SCM_ and increase of T_CM_ and T_EM_ in the spleen suggested that T_SCM_ could be quickly differentiated into T_CM_, T_EM_ and effector cells. Enriched CD8^+^ T_CM_ confers a potent *in vivo* antitumor immune response leading to sustaining long-term tumor regression in melanoma model.^32^ From day 14–28 after the treatment, T_SCM_ was increased, and CD8^+^ T cells displayed a phenotype of self-renewing memory T cells from day 21–90, suggesting that the majority of memory CD8^+^ T cells was likely T_SCM_ to ensure durable protection against tumor.

Early studies generally focus on direct tumor recognition by CD8^+^ T cells,^33, 34^ whereas recently developed models have used CD4^+^ T cells.^35^ CD4^+^ T cells could acquire direct cytolytic activity.^36, 37^ Moreover, the presence of CD4^+^ T cells is necessary for clonal expansion upon re-encountering antigen during antigen-specific CD8^+^ cytotoxic T-lymphocyte (CTL) priming.^29^ If lacking CD4^+^ T cells help, memory CD8^+^ T cells can become functionally impaired.^38^ In this study, Tregs subset was diminished, whereas CD4-CTL, Th1, Th2, Th17 and Tfh subsets were predominated in CD4^+^ T cells at the different stage after the treatment, which indicated that CD4^+^ antitumor immune response induced by the treatment overwhelmingly overcame to its immunosuppressive function. In the late stage of the treatment, CD4^+^ T cells in the treated mice were differentiated into predominant Th1, CD4^+^ CTL and Tfh subsets, which could maintain long-term antitumor immunity. By far, hyperthermia, therapeutic vaccines (dendritic cell vaccine) and antitumor mAb treatment are shown to mainly drive differentiation of CD4^+^ T cells into Th1 cells,^39, 40, 41, 42^ whereas multiple CD4^+^ T-cell lineages induced by the therapeutic strategies participated in antitumor immunity has not been reported. This study revealed that multiple CD4^+^ T-cell lineages differentiation was significantly dominated at the different stage after the treatment, which would be a characteristic hallmark of long-term CD4^+^ T-cell immunity triggered by the treatment. The precise mechanism of CD4^+^ T-cell differentiation toward immunostimulatory lineages needs to be further studied.

Clinically, standard treatment for melanoma patients is surgery followed by adjuvant therapy. Adjuvant therapy with IFN is associated with a significant improvement in disease-free survival (DFS) and overall survival (OS).^43, 44^ In our study, without any adjuvant therapy, a high level of IFN-*γ* was boosted after the cryo-thermal therapy, which provides an ideal microenvironment for optional T-cell function.^30^ Altogether, our studies demonstrated that long-term survival of a murine B16F10 melanoma induced by the cryo-thermal therapy mainly attributed to the induction of antigen-specific CD4^+^ T cells maintaining melanoma-specific memory immune response.

## Conclusion

Our study demonstrated that primary melanoma treated by the cryo-thermal therapy produced long-term tumor-free survival that was dependent on CD4^+^ T-cell orchestrating antitumor immune memory response. Further study would be required to determine the mechanism of durable antitumor immune response in multiple CD4^+^ T-cell subsets. The cryo-thermal therapy could be developed as a new modality to prevent tumor recurrence and metastasis.

## Materials and Methods

### Animal model

The female C57BL/6 mice were obtained from Shanghai Slaccas Experimental Animal Co., Ltd. (China) and used for experimental study at the age of 6–8 weeks. They were housed in isolated cages and a 12 h light/dark cycle environment, feeding with sterile food and acidified water with pH value kept at 2.5–2.8. All animal experiments were approved by the Animal Welfare Committee of Shanghai Jiao Tong University and experimental methods were performed in accordance with the guidelines of Shanghai Jiao Tong University Animal Care (approved by Shanghai Jiao Tong University Scientific Ethics Committee). Murine B16F10 cells (donated by Professor Weihai Yin at Med-X Research Institute, Shanghai Jiao Tong University) were cultured in RPMI 1640 medium (Hyclone, USA) supplemented with 10% FBS, plus 100 U/ml penicillin and 100 g/ml streptomycin (Shanghai Sangon, China). To prepare the tumor-bearing mice, approximately 5 × 10^5^ cells were injected subcutaneously into the right femoral region of each mouse. The tumor sizes were measured every 2–3 days and its volume was estimated using the following formula: *V* (cm^3^)=π × *L* (major axis) × *W* (minor axis) × *H* (vertical axis)/6.

### The thermal therapy procedures

The system developed in our laboratory was composed of liquid nitrogen for cooling and radiofrequency (RF) for heating.^45^ To reduce the effect of contact thermal resistance and obtain a constant thermal delivery during the treatment, a probe was designed with a cylinder-shaped tip of 10 mm in diameter for the thermal therapy of subcutaneous tumor.^46^ Subcutaneous injection of B16F10 melanoma cells into C57BL/6 mice leads to form primary tumors in 7–9 days and spontaneous metastasis in lungs.^23^ Twelve days after tumor inoculation, when the tumor volume reached about 0.2 cm^3^, the mice were divided into two groups: tumor-bearing group without the treatment (control), the cryo-thermal group with freezing at the temperature of −20 °C for 5 min followed by RF heating at the temperature of 50 °C (the simulated temperature distribution during the RF heating process was performed previously^22^) for 10 min on primary tumor (the cryo-thermal). The mice were anesthetized with intraperitoneal (i.p.) injection of 1.6% pentobarbital sodium (0.5 ml/100 g, Sigma-Aldrich, St. Louis, MO, USA). The tumor site was sanitized with alcohol and iodine tincture before the treatment. All the procedures were performed aseptically. In five independent trials (*n*=37 for control or the cryo-thermal group, respectively), the survival time and survival rate were determined, respectively.

### Pathological observation

Subcutaneous injection of B16F10 melanoma cells into C57BL/6 mice leads to form primary tumors in 7–9 days and spontaneous micrometastasis in lungs.^23^ Because macroscopic tumor nodules were not visible at the early stage through subcutaneous injection of B16F10 melanoma cells, hematoxylin and eosin (H&E) staining was used to observe micrometastasis in lungs before and after the cryo-thermal therapy. On day 12, 26 and 102 after tumor inoculation, three mice were randomly selected from each group (on day 102 after tumor inoculation, all mice in the control group were dead) and killed by an intraperitoneal injection of pentobarbital sodium (Sigma-Aldrich). The lung tissues were removed, fixed in 10% formalin, and paraffin-embedded sections were prepared. Paraffin-embedded tissues were sliced into sections of 7 *μ*m each. Hematoxylin and eosin staining of lung tissue was performed and the pathological changes were visualized under a microscope (Nikon Eclipse Ci-E, Nikon, Beijing, China).

### Preparation of a single-cell suspension and FACS analysis

The mice were killed on day 7, 14, 21, 28, 90 and 180 after the cryo-thermal therapy, and the spleen, lung tissues and peripheral blood were collected (*n*=3 per group at each time point). Single-cell suspension of splenocytes was prepared using GentleMACS dissociator (Miltenyi Biotec, Bergisch Gladbach, Germany), and then treated with erythrocyte-lysing reagent containing 0.15 M NH_4_Cl, 1.0 M KHCO_3_ and 0.1 mM Na_2_EDTA to remove the red blood cells. The single-cell suspension from lung tissue was prepared using the GentleMACS single-cell isolation protocol (Miltenyi Biotec). Briefly, the lung tissues were minced and digested in PBS containing 0.1% collagenase (Sigma-Aldrich), 0.01% hyaluronidase (Sigma-Aldrich) and 0.002% DNase I (Promega) for 40 min at 37 °C. The cells were dispersed using 70 *μ*m mesh screens and the red blood cells were lysed by using erythrocyte-lysing reagent. The peripheral blood samples were treated with erythrocyte-lysing reagent, washed and resuspended in PBS. These cells were stained to characterize immune cell populations using fluorescence conjugated antibodies binding cell-specific surface marker for 30 min at 4 °C. The staining antibodies including CD11b-FITC (clone M1/70), Gr-1-PE (clone RB6-8C5), CD3-FITC (clone 145-2C11), CD3-PerCP/cy5.5 (clone 145-2C11), CD4-PE (clone GK1.5), CD25-FITC (clone 3C7), CD8-APC (clone 53-6.7), CD44-PE (clone IM7) and CD62L-PE-Cy7 (clone MEL-14) were purchased from eBioscience (Santiago, CA, USA). A total 1 × 10^4^ events were collected for all analyses using the BD FACS Aria II cytometer (BD Biosciences) and the data were analyzed using FlowJo software.

### T-cell isolation

For the isolation of T cells, the spleens from the treated and tumor-bearing C57BL/6 mice were harvested on day 7, 14, 21, 28 and 90 after the treatment (*n*=3 mice per group at each time point), and prepared using GentleMACS dissociator (Miltenyi Biotec). The CD4^+^ and CD8^+^ T cells were purified from splenocytes using Easysep mouse CD4^+^ and CD8^+^ T cell Enrichment Kits (StemCell Technologies, Vancouver, BC, Canada) according to the manufacturer's instructions. The CD4^+^ and CD8^+^ T cells with a purity of >90% were used for experiments.

### RNA isolation and real-time PCR

Total RNA was prepared from purified CD4^+^ and CD8^+^ T cells using TRIzol Reagent (TaKaRa, Dalian, China). Absorbance at 260/280 nm for mRNA purity at a ratio above 1.9 was achieved for all the samples used. cDNA was made using a PrimeScript RT reagent kit (TaKaRa). Quantitative real-time PCR was performed on ABI 7900HT sequence detection system, and SDS software (Applied Biosystems, Foster City, CA, USA) using SYBR Premix Ex Taq (TaKaRa) and samples were amplified in 384-well plates. Because most of the tumor-bearing mice died in 30 days after tumor inoculation, the mRNA in splenic CD4^+^ and CD8^+^ T cells from tumor-bearing mice on day 26 after tumor inoculation was used as the control to compare the marker profiles of splenic CD4^+^ and CD8^+^ T cells from the treated mice, expressed as a fold difference. The same control tumor-bearing mice (on day 26 after tumor inoculation) were used for all the four time points. Markers of CD4^+^ T-cell sub-lineages were included in the following: IFN-*γ*, T-bet, IL-2, IL-12, TNF-*α* (for Th1 cells); IL-5, IL-4, IL-13, GATA3 (for Th2 cells); TGF-*β*, IL-10, FoxP3 (for Tregs); IL-17A, ROR*γ*t, CCL20 (for Th17 cells); Perforin, GzmB, Eomes, IFN-*γ* (for cytolytic CD4^+^ T cells (CD4^+^CTL cells)); Bcl-6 and IL-21 (for Tfh cells).^26^ The primer sequences of mouse genes presented in Supplementary Table 1. Relative expression levels of mRNA for each gene were normalized to GAPDH determined by using the Ct value and assessed using relative quantification (delta–delta Ct method). All the experiments were performed in triplicates.

### Depletion of CD4^+^ and CD8^+^ T-cell subsets *in vivo*

For T-cell-depleting experiments, the treated mice (*n*=6 mice per group) after 45 days were depleted with anti-CD4 or anti-CD8 monoclonal antibodies, respectively (mice were injected i.p. with mAb against CD4 or CD8, 1 day before tumor re-challenge and 2 days after tumor re-challenge, then mice received depletion antibodies every 5 days for 4 weeks to maintain the depleted condition), then the treated mice after CD4^+^ or CD8^+^ lymphocyte depletion was re-challenged with 1 × 10^5^ B16F10 cells. The age-matched naive mice were inoculated with 1 × 10^5^ B16F10 cells as control (tumor-bearing) group. The potential effect of Ab depletion was confirmed *in vivo*. The tumor size was monitored every day.

### Enzyme-linked immunosorbent assay

One milliliter of blood was obtained from mice on day 14 and 90 after the treatment. The specimens were centrifuged and the serum was collected. Then the serum was analyzed for IFN-*γ* using commercial enzyme-linked immunosorbent assay (ELISA) kits from Boster Biotech, Shanghai, China. A standard curve was established according to the manufacturer's instruction. The experimental values were computed with the use of regression analysis. Normal mice and tumor-bearing mice were used as control, six mice were used for each group.

### Tumor re-challenge analysis

On day 45 after the cryo-thermal therapy, the mice were re-challenged by subcutaneous injection in the left femoral region with 1 × 105 B16F10 cells (*n*=3 per group). To investigate the distal effect of the treatment, primary B16F10 melanoma tumor in C57BL/6 mice were treated, and 45 days later, the treated mice were intravenously infused with 1 × 103 B16F10 melanoma cells and lung metastasis were enumerated (*n*=3 per group). Another group of the treated mice after 45 days were re-challenged subcutaneously with 1 × 10^5^ B16F10 cells on the right flank and 1 × 10^6^ Gl261 cells on the left flank (*n*=6 per group). Furthermore, the experiment was also performed on day 60 to add one more time point after the treatment as mentioned above, to rule out the elicited local antitumor immunity. The same batch of healthy mice were inoculated with 1 × 10^5^B16F10 cells on the left and 1 × 10^6^ GL261 cells on the right and was used as control (tumor-bearing) group (*n*=6 per group). Tumor growth was measured every day using calipers, and the tumor volumes were estimated.

### Statistical analysis

The Student's *t*-test and one-way ANOVA were used for statistical comparisons using Graph Pad Prism 6. Figures denoted statistical significance of *P<0.05, **P<0.01 and ***P<0.001. *P*-values <0.05 was considered to be statistically significant. Flank tumor growth curves were analyzed using two-way ANOVA. To assess the survival differences, Kaplan–Meier curves were produced and analyzed by log-rank tests. The results were expressed as mean±S.D.
