# Supplementary material for: The cryo-thermal therapy eradicated melanoma in mice by eliciting CD4+ T-cell-mediated antitumor memory immune response
Source: Cell Death Dis. 2017 Mar 23;8(3):e2703–. doi: 10.1038/cddis.2017.125 (PMC5386530; doi:10.1038/cddis.2017.125)
Supplement: Supplementary Information [file cddis2017125x1.docx]

**Supplementary table 1.** Primer sequences of various genes in this study.

Table 1. Primer sequences of various genes studied.

| **Name** | **Primer Sequence (5´-3´)** | **Gene (size)** | **Annealing temperature** |
| --- | --- | --- | --- |
| IFN-γ-forward | ATGAACGCTACACACTGCATC | 182 bp. | 60℃ |
| IFN-γ-reverse | CCATCCTTTTGCCAGTTCCTC |  |  |
| Tbet-forward | GTTCAACCAGCACCAGACAGAG | 135 bp. | 60℃ |
| Tbet-reverse | TGGTCCACCAAGACCACATC |  |  |
| IL-2-forward | TGAGCAGGATGGAGAATTACAGG | 120 bp. | 60℃ |
| IL-2-reverse | GTCCAAGTTCATCTTCTAGGCAC |  |  |
| IL-12-forward | TGGTTTGCCATCGTTTTGCTG | 123 bp. | 60℃ |
| IL-12-reverse | ACAGGTGAGGTTCACTGTTTCT |  |  |
| TNFα- forward | TTCTGTCTACTGAACTTCGGGGTGATCGGTCC | 306 bp. | 60℃ |
| TNFα-reverse | GTATGAGATAGCAAATCGGCTGACGGTGTGGG |  |  |
| IL-5-forward | TCAGGGGCTAGACATACTGAAG | 168 bp. | 60℃ |
| IL-5-reverse | CCAAGGAACTCTTGCAGGTAAT |  |  |
| IL-4- forward | CCCCAGCTAGTTGTCATCCTG | 78 bp. | 60℃ |
| IL-4-reverse | CAAGTGATTTTTGTCGCATCCG |  |  |
| IL-13- forward | CGGCAGCATGGTATGGAGTG | 85 bp. | 60℃ |
| IL-13-reverse | ATTGCAATTGGAGATGTTGGTCAG |  |  |
| GATA-3- forward | GGATGTAAGTCGAGGCCCAAG | 117 bp. | 60℃ |
| GATA-3-reverse | ATTGCAAAGGTAGTGCCCGGTA |  |  |
| TGF-β-forward | CTCCCGTGGCTTCTAGTGC | 105 bp. | 60℃ |
| TGF-β-reverse | GCCTTAGTTTGGACAGGATCTG |  |  |
| IL-10- forward | GCTCTTACTGACTGGCATGAG | 105 bp. | 60℃ |
| IL-10-reverse | CGCAGCTCTAGGAGCATGTG |  |  |
| Foxp3- forward | AGCAGTGTGGACCGTAGATGA | 77 bp. | 60℃ |
| Foxp3-reverse | GGCAGGGATTGGAGCACTT |  |  |
| IL-17A- forward | GAAGGCCCTCAGACTACCTCAA | 150 bp. | 60℃ |
| IL-17A- reverse | TCATGTGGTGGTCCAGCTTTC |  |  |
| IL-21- forward | CAGGCTAAGAGCTTGTATCGTTTGG | 248 bp | 60℃ |
| IL-21- reverse | AGGACTGGCTGAGTCTTGAGCAC |  |  |
| RORrt- forward | CGCGGAGCAGACACACTTA | 167 bp | 60℃ |
| RORrt- reverse | CCCTGGACCTCTGTTTTGGC |  |  |
| CCL20- forward | TACAGACGCCTCTTCCTTCCA | 147 bp | 60℃ |
| CCL20- reverse | CAGCCCTTTTCACCCAGTTC |  |  |
| perforin- forward | CTGCCACTCGGTCAGAATG | 88 bp | 60℃ |
| perforin- reverse | CGGAGGGTAGTCACATCCAT |  |  |
| GzmB- forward | CCACTCTCGACCCTACATGG | 142 bp | 60℃ |
| GzmB- reverse | GGCCCCCAAAGTGACATTTATT |  |  |
| Eomes- forward | GGCCCCTATGGCTCAAATTCC | 62 bp | 60℃ |
| Eomes- reverse | CCTGCCCTGTTTGGTGATG |  |  |
| Bcl-6- forward | ATGTACAGCCATCTCCCGCT | 134 bp | 60℃ |
| Bcl-6- reverse | TTAGGGACTTGCCTGGCACT |  |  |
| sca-1- forward | AGGAGGCAGCAGTTATTGTGG | 114 bp | 60℃ |
| sca-1- reverse | CGTTGACCTTAGTACCCAGGA |  |  |
| GAPDH- forward | AGGTCGGTGTGAACGGATTTG | 98 bp | 60℃ |
| GAPDH- reverse | GGGGTCGTTGATGGCAACA |  |  |
