# Supplementary figures and images for: The cryo-thermal therapy eradicated melanoma in mice by eliciting CD4+ T-cell-mediated antitumor memory immune response
Source: Cell Death Dis. 2017 Mar 23;8(3):e2703–. doi: 10.1038/cddis.2017.125 (PMC5386530; doi:10.1038/cddis.2017.125)

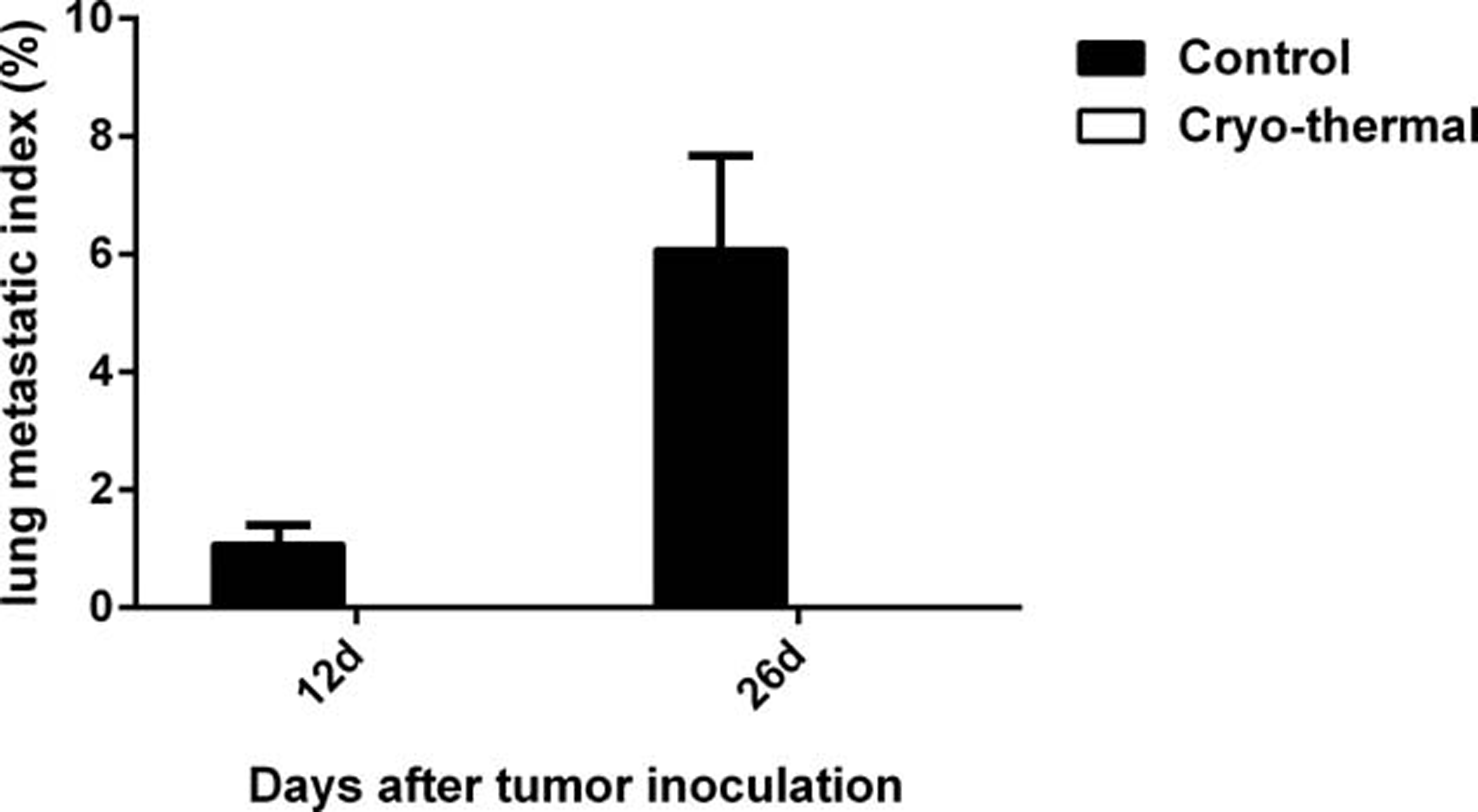

Supplement: Supplementary Figure 1 [file cddis2017125x2.tif]

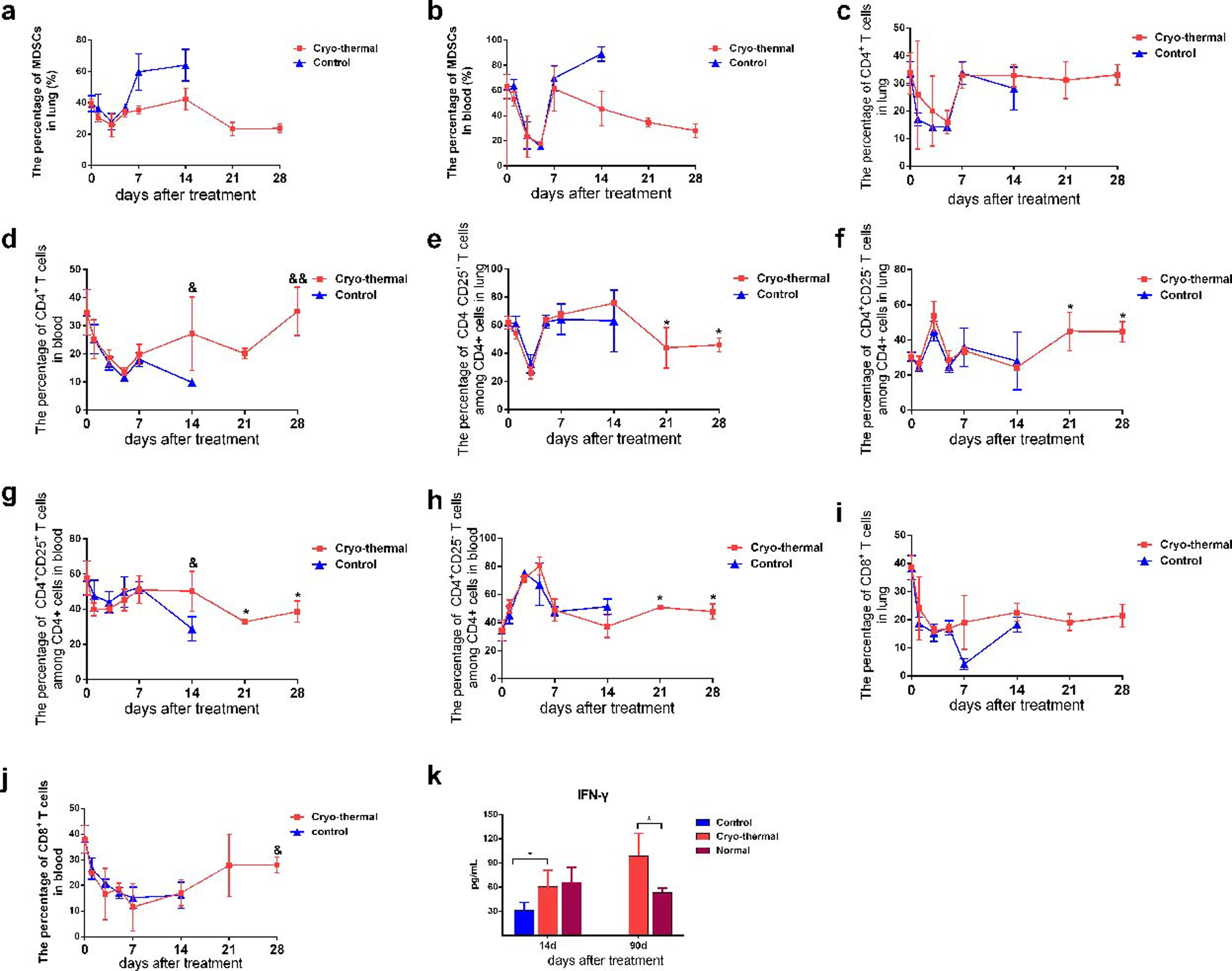

Supplement: Supplementary Figure 2 [file cddis2017125x3.tif]

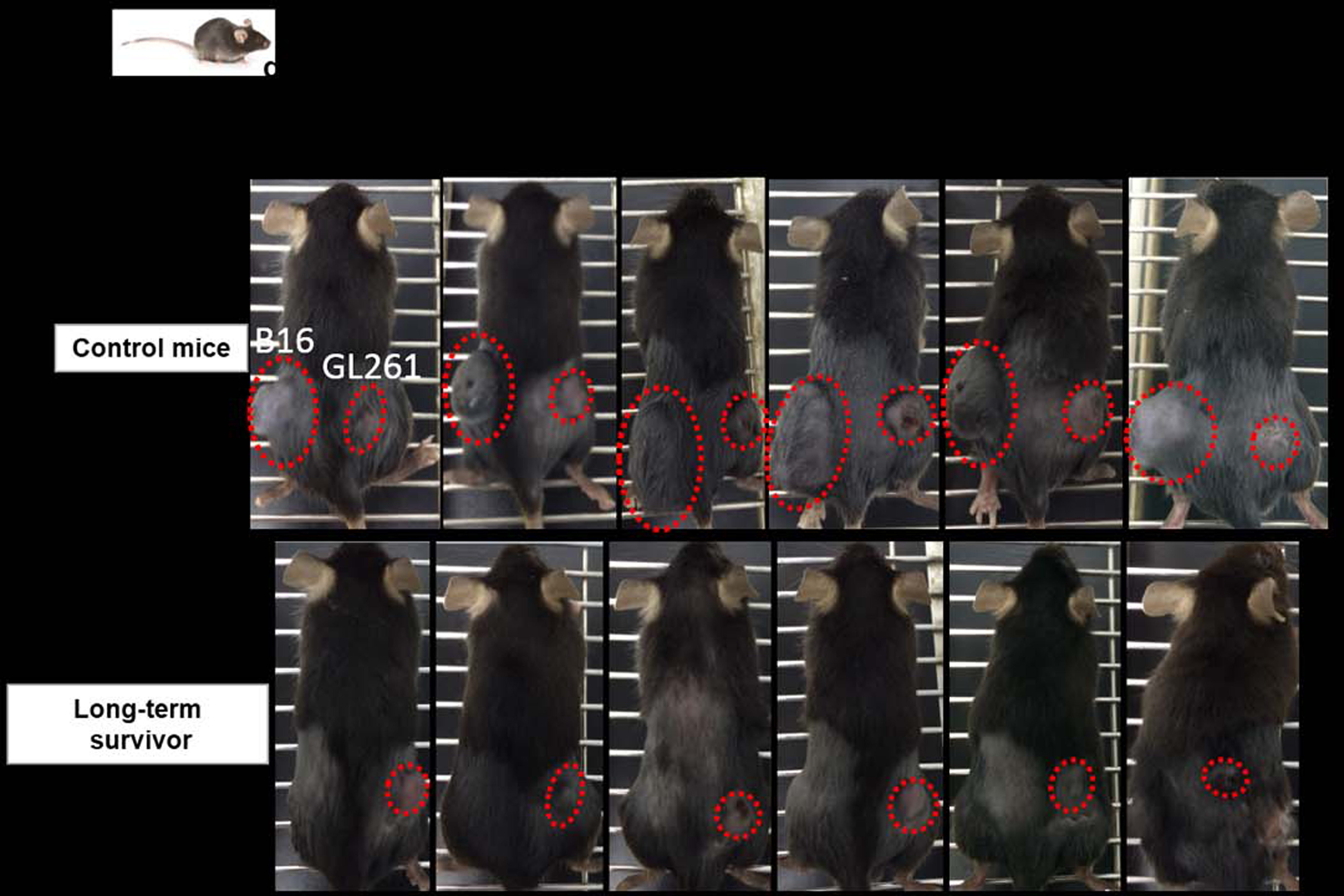

Supplement: Supplementary Figure 3 [file cddis2017125x4.tif]
